# TRACING THE FOOTSTEPS OF THE SCHOLARS FROM DE TORNYAY CENTER FOR HEALTHY AGING AT UNIVERSITY OF WASHINGTON

**DOI:** 10.1093/geroni/igae098.2943

**Published:** 2024-12-31

**Authors:** Yanjing Liang, Priscilla Carmiol-Rodriguez, Sarah McKiddy, Paige Bartlett, Heather Wicklein Sanchez, Basia Belza

**Affiliations:** University of Washington, Seattle, Washington, United States; University of Washington, Washington, United States; University of Washington, Seattle, Washington, United States; University of Washington, Seattle, Washington, United States; University of Washington, Seattle, Washington, United States; University of Washington, Seattle, Washington, United States

## Abstract

The de Tornyay Center for Healthy Aging has been pivotal in advancing research, education, and practice since 1998. The center faculty has mentored over 165 students, connected them to community partners, awarded them over $757,000 in research funding, and supported the dissemination of their work through travel scholarships. This paper presents the evolution of the scholar community by showcasing scholars’ contributions over time. An online survey was distributed to 87 former de Tornyay Center scholars, resulting in 32 responses. Results indicate that after graduating scholars pursued careers in practice (n=21, 65.6%), teaching (n=18, 56.2%), and research (n=13, 40.6%). Contributions include publishing in high-impact journals, securing research funding from institutions like the National Institutes of Health, and pioneering innovative practice models. Scholars demonstrated leadership in the COVID-19 response and made impactful changes in clinical settings. Scholars have engaged in shaping the next generation of nursing professionals through teaching, developing educational models, and contributing to professional service globally. Scholars credited the de Tornyay Center with boosting their research skills (n=24, 75%) and providing crucial career guidance and mentorship (n=24, 75%). Early involvement in scientific research at the center influenced students’ academic paths, fostering a commitment to gerontological nursing. These findings contribute to understanding scholars’ journeys and form a foundation for shaping the center’s strategic plan. The results underscore the center’s role in nurturing scholars who significantly contribute to healthcare, cultivating a talented workforce, and influencing scholars’ career paths. The de Tornyay scholar program is instrumental in fostering professionalism in scholars’ careers.

